# Unveiling the Influence of Water Molecules for NF_3_ Removal by the Reaction of NF_3_ with OH: A DFT Study

**DOI:** 10.3390/molecules29174033

**Published:** 2024-08-26

**Authors:** Jiaxin Liu, Yong Zhao, Xueqi Lian, Dongdong Li, Xueling Zhang, Jun Chen, Bin Deng, Xiaobing Lan, Youxiang Shao

**Affiliations:** 1 Hunan Provincial Key Laboratory of Xiangnan Rare-Precious Metals Compounds Research and Application, School of Chemistry and Environmental Science, Xiangnan University, Chenzhou 423000, China; 18573582931@163.com (J.L.); 19973466638@163.com (Y.Z.); dongdong_live168@163.com (D.L.); jchen4174@xnu.edu.cn (J.C.); dengbinxnu@163.com (B.D.); 2 Key Laboratory of Electronic Functional Materials and Devices of Guangdong Province, School of Chemistry and Materials Engineering, Huizhou University, Huizhou 516007, China; 2006070302129@stu.hzu.edu.cn (X.L.); zxl@ahnu.edu.cn (X.Z.)

**Keywords:** NF_3_ removal, OH radical, water molecules, mechanism, DFT calculation

## Abstract

The removal of nitrogen trifluoride (NF_3_) is of significant importance in atmospheric chemistry, as NF_3_ is an important anthropogenic greenhouse gas. However, the radical species OH and O(^1^D) in atmospheric conditions are nonreactive towards NF_3_. It is necessary to explore possible ways to remove NF_3_ in atmosphere. Therefore, the participation of water molecules in the reaction of NF_3_ with OH was discussed, as water is abundant in the atmosphere and can form very stable complexes due to its ability to act as both a hydrogen bond donor and acceptor. Systemic DFT calculations carried out at the CBS-QB3 and ωB97XD/aug-cc-pVTZ level of theory suggest that water molecules could affect the NF_3_ + OH reaction as well. The energy barrier of the S_N_2 mechanism was decreased by 8.52 kcal/mol and 10.58 kcal/mol with the assistance of H_2_O and (H_2_O)_2_, respectively. Moreover, the presence of (H_2_O)_2_ not only reduced the energy barrier of the reaction, but also changed the product channels, i.e., formation of NF_2_O + (H_2_O)_2_-HF instead of NF_2_OH + (H_2_O)_2_-F. Therefore, the removal of NF_3_ by reaction with OH is possible in the presence of water molecules. The results presented in this study should provide useful information on the atmospheric chemistry of NF_3_.

## 1. Introduction

As the most extensively used perfluoro compound, nitrogen trifluoride (NF_3_) has attracted great interest in recent years. NF_3_ is commonly used in the semiconductor industry [1,2,3] and as a fluorine-supplying source in the electronic industry [4,5]. The industrial use of NF_3_ had been considered safe for a long time, as it did not produce carbon contamination residues. Hence, the production of NF_3_ as a substitute for other perfluorinated gases such as CF_4_ and C_2_F_6_ increased dramatically in recent years [6], resulting in a very large amount of NF_3_ atmospheric emissions. Unfortunately, recent studies have warned that there is a clear risk in using NF_3_ [7]. Firstly, NF_3_ is considered a new greenhouse gas, although it is not included in the list of greenhouse gases in the Kyoto protocol [8,9]. In fact, NF_3_ has a global warming potential (GWP) of 17,200, which is 10,800 times greater than that of CO_2_ when compared over a 100-year period [10,11,12]. Furthermore, NF_3_ and its decomposition products have been proposed to be toxic and pose a health risk [8]. Because of these concerns, great interest has focused on developing new processes to destroy or remove unreacted effluent NF_3_.

To date, various methods have been reported for the adsorption and decomposition of NF_3_ [13,14,15,16,17,18]. However, these methods are designed to deal with the tail gas in the semiconductor industry and electronic industry. On the other hand, there is a significant shortage of research on removal and decomposition processes for NF_3_ in the atmosphere. Gargano et al. [19] studied two important reactions involved in the decomposition of NF_3_, i.e., NF_3_ + F and N_2_ + F. Later, Cunha and coworkers [20] investigated other reactions involved in the decomposition of NF_3_ employing theoretical calculations at the CCSD(T)/cc-pVTZ level of theory, for example, NF_2_ + N, NF_3_ + NF, and the dissociation of N_2_F_4_ and N_2_F_3_. These pioneer studies provide fundamental insight into the mechanism of NF_3_ decomposition.

Several studies have also focused on the removal of NF_3_ in the atmosphere through the reaction with atmospheric oxidants. Wine and coworkers [21] studied the reaction of NF_3_ with O(^1^D), measured the rate coefficient to be k(T) = 2.0 × 10^−11^ exp(52/T) cm^3^ molecule^−1^s^−1^, and suggested that the reaction with O(^1^D) is an important atmospheric sink for NF_3_. Baasandorj and coworkers [22] also measured the rate coefficient of O(^1^D) with NF_3_, which is in good agreement with the results of Wine and coworkers. However, the reaction of reactive OH radical with NF_3_ was not mentioned. Dillon and coworkers [23] explored the possibility of removing NF_3_ by reactions with the atmospheric oxidants O(^1^D), OH and O_3_, and the results showed that the reaction rate of NF_3_ + OH is as slow as 2.0 × 10^−29^ cm^3^ molecule^−1^s^−1^; thus, they concluded that OH could not play an important role in atmospheric NF_3_ degradation. Although the reaction of OH with NF_3_ is extremely slow, the possibility of removing NF_3_ by reaction with OH should not be excluded because water molecules in the atmosphere have been shown to have a significant chemical catalytic effect on certain atmospheric reactions. Buszek and coworkers [24] reviewed the effect of water molecules on various atmospheric reactions, including radical–molecule, radical–radical, molecule–molecule and unimolecular reactions. It is surprising that, to our best knowledge, the influence of water molecules on the reaction of OH + NF_3_ has not been explored yet, though the reaction of OH with various molecules, such as HCOOH [25], HNO_3_ [26],CH_3_CHO [27,28,29], fluoroalcohols [30], HOCl [31], glyoxal [32,33], CH_4_ [34], DMSO [35], CH_3_OH [36,37,38], etc., have been studied extensively. Could additional water molecules accelerate the reaction of OH + NF_3_? If the answer is positive, how do the additional water molecules affect the reaction? Here, we decided to study the reaction of OH + NF_3_ with the participation of water molecules by using computational methods. These questions are critical for exploring the processes of removing NF_3_ in the atmosphere.

## 2. Results and Discussion

In principle, it is better to carry out benchmark calculations aiming to assess the accuracy of the DFT methods. Fortunately, several references have proved that the ωB97XD functional including dispersion was capable of treating various reactions [39,40]. As a result, all the discussions are based on the data obtained from CBS-QB3//ωB97XD/aug-cc-pVTZ methods. Moreover, as the reactions discussed here are in the gas phase, the electronic energy with zero-point energy was employed to discuss the thermodynamics [41,42].

### 2.1. The Reaction of NF_3_ + OH

**Figure 1:**
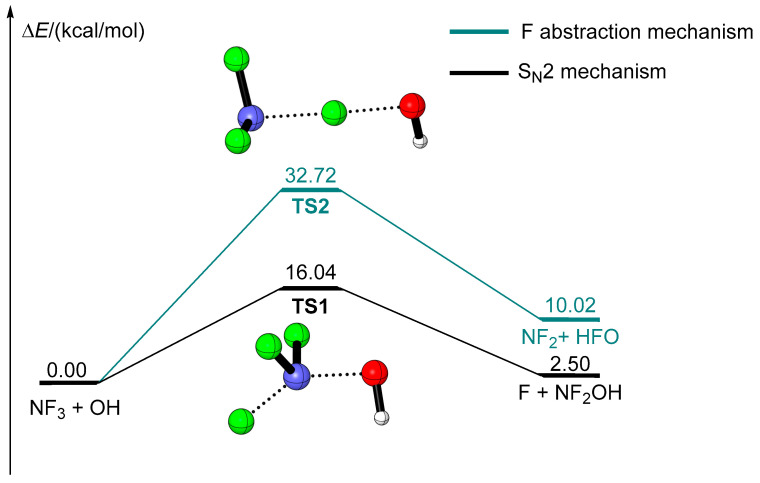
The energy profile of the NF_3_ + OH reaction.

According to a previous work [22], the reaction of NF_3_ + OH can be carried out through three distinct processes, i.e., the S_N_2 mechanism, F abstraction and H addition to the N center. However, the energy barrier is too high for H addition to N to be of consideration. As a result, only the S_N_2 mechanism and F abstraction were discussed here. As for the S_N_2 mechanism, an OH radical attacks the N, while one N-F bond is broken simultaneously, forming NF_2_OH and F. As shown in Figure 1, the corresponding transition state is **TS1** with an energy barrier of 16.04 kcal/mol. Unfortunately, the product is endothermic by 2.50 kcal/mol, indicating this process is unfavorable thermodynamically, especially in atmospheric conditions. In the case of the F abstraction mechanism, an OH radical abstracts F from NF_3_ directly, leading to the production of HFO and NF_2_. The transition state for this process is **TS2**, in which the OH interacts with the leaving F. As can be seen in Figure 1, the energy barrier of **TS2** (32.72 kcal/mol) is much higher than that of **TS1**. Moreover, the product is endothermic by as much as 10.02 kcal/mol. These results indicate the F abstraction process is unfeasible. In a word, although the S_N_2 mechanism is predominant in comparison to the F abstraction mechanism, the reaction of NF_3_ + OH is difficult to accomplish in view of thermodynamics. This is in good accordance with the extremely slow reaction rate measured experimentally [23]. As a result, the removal of NF_3_ through the gas-phase reaction with OH radial is of minor importance in atmospheric conditions. These results are in accordance with a previous report [23].

### 2.2. The Influence of Water Molecules on the Reaction of NF_3_ + OH

**Figure 2:**
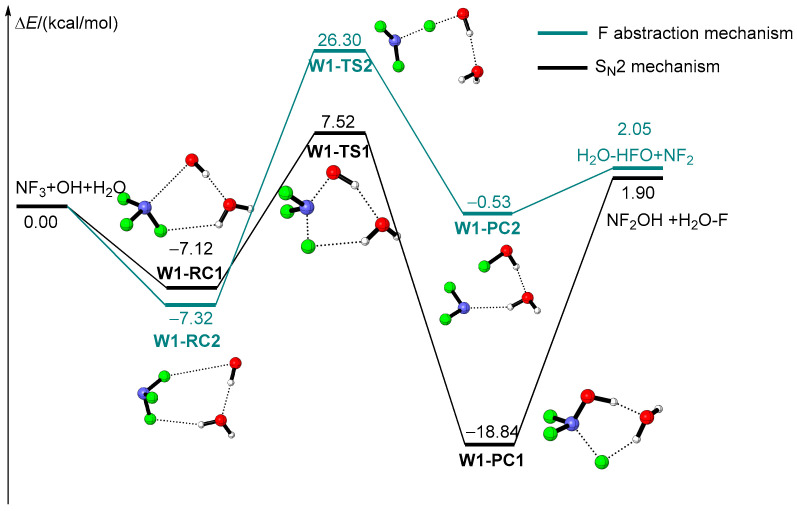
Energy profiles of one additional H_2_O participating in the reaction of NF_3_ + OH.

Inspired by the fact that the participation of water molecules could affect various atmospheric reactions in the gas phase [43,44,45,46,47,48,49], the influence of water molecules on the reaction of NF_3_ + OH is discussed here. In the condition where one H_2_O participates in the reaction, the NF_3_ + OH reaction takes place through two distinct process similar to the naked reaction shown in Figure 2. The corresponding geometry structures of all intermediates and transition states are available in Figure S1.

In contrast to the naked reaction, a pre-reactive complex formed in the entrance of the reaction due to the existence of a hydrogen bond between H_2_O and the reactants. For example, **W1-RC1** and **W1-RC2** are the pre-reactive complexes for the S_N_2 mechanism and F abstraction mechanism, respectively, as shown in Figure 2. Attributed to the formation of a hydrogen bond, **W1-RC1** and **W1-RC2** are 7.12 kcal/mol and 7.32 kcal/mol more stable than the reactants, respectively. Starting from **W1-RC1**, the reaction takes place via the S_N_2 mechanism. The corresponding transition state is **W1-TS1**. It should be noted that the structure of **W1-TS1** is similar to that of **TS1,** except the breaking F atom bonded to the H_2_O due to the formation of an F∙∙∙H∙∙∙O hydrogen bond. The energy barrier of **W1-TS1** is 14.64 kcal/mol, which is only 1.40 kcal/mol lower than that of **TS1**. Therefore, the influence of one H_2_O molecule on the S_N_2 mechanism is negligible in view of the kinetics. On the other hand, although the product complex **W1-PC1** was located 18.84 kcal/mol below the reactants owing to the formation of a hydrogen bond, the final product, NF_2_OH + H_2_O-F, is endothermic by 1.90 kcal/mol, which is similar to that of **PC1**. It is reasonable to conclude that an additional H_2_O molecule is of no influence on the S_N_2 mechanism in view of the thermodynamics as well. As for the F abstraction mechanism, the corresponding transition state is **W1-TS2**, with a geometry structure similar to that of **TS2**. Unfortunately, the relative energy of **W1-TS2** is as high as 26.30 kcal/mol. As a result, the reaction should overcome the energy barrier of 33.62 kcal/mol, which is even about 1 kcal/mol larger than that of F abstraction in the absence of H_2_O (**TS2**). Thus the participation of one H_2_O molecule is unable to accelerate the F abstraction process. In a word, when one additional H_2_O molecule takes part in the reaction of NF_3_ + OH, neither the kinetics nor thermodynamics are affected. This can be explained by the structure of the transition states. As can be seen in Figure S1, the hydrogen transfer process is not involved in either the S_N_2 mechanism or F abstraction mechanism. The additional H_2_O molecule only connects OH and F with the formation of a hydrogen bond rather than assisting the hydrogen transfer. Thus an additional, single H_2_O molecule only acts as a spectator rather than catalyst in the reaction of NF_3_ + OH. It is not unexpected that the effects of H_2_O molecules on various atmospheric reactions reported in the references do not appear here.

### 2.3. The Influence of an Additional Two H_2_O Molecules on the NF_3_ + OH Reaction

**Figure 3:**
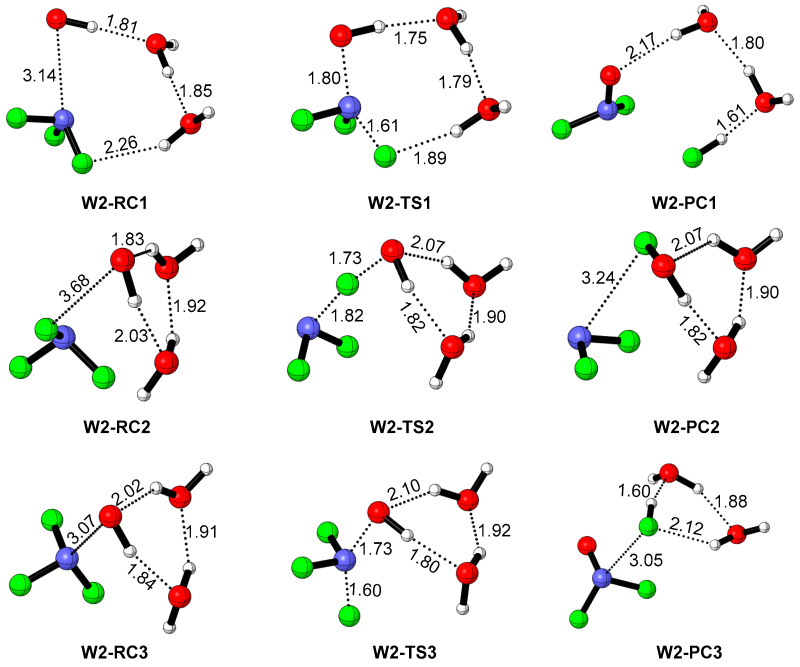
The structures of intermediates and transition states involved in NF_3_ + OH reactions with two H_2_O molecules participating. The distances are in Å.

**Figure 4:**
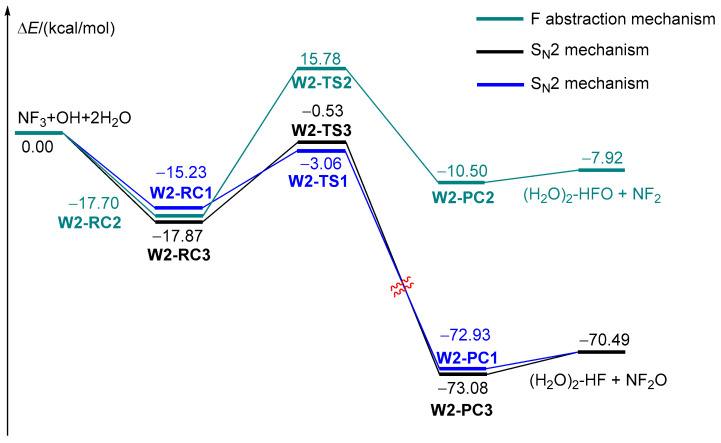
The energy profiles of NF_3_ + OH reactions with an additional two H_2_O molecules.

The structures of various pre-reactive complexes and transition states for the NF_3_ + OH reaction with an additional two H_2_O are shown in Figure 3. In contrast to the reaction with one participating H_2_O, there are three possible processes, as can be seen from the corresponding potential energy profiles (see Figure 4).

For the S_N_2 mechanism, the structure of the pre-reactive complex **W2-RC1** is extremely similar to that of **W1-RC1**. However, the F-H bond in **W2-RC1** is 0.3 Å shorter than that of **W1-RC1**, and the distance of N-O is reduced by about 0.2 Å. This could be attributable to the stabilization energy provided by the hydrogen bond of two H_2_O molecules, and could be proved by the energy of **W2-RC1**, which is 8 kcal/mol more stable than that of **W1-RC1**. The transition state corresponding to the broken N-F bond and N-O bond formation is **W2-TS1,** with an energy barrier of 12.17 kcal/mol. Moreover, **W2-TS1** is located 3.06 kcal/mol below the initial reactants; thus, this transformation is accessible kinetically. It should be attributed to the direct participation of (H_2_O)_2_ in the reaction as a proton shuttle. As shown in Figure 3, the F-H in the **W2-TS1** bond has shrunk to 1.89 Å, which is 0.32 Å shorter than that of **W1-TS1**, indicating the eliminated F has connected to the H_2_O molecules. This is verified by the intrinsic reaction coordinate (IRC) [50] calculation (see Figure S2). Moreover, the O-H bond in the OH radical is intended to break in the product direction of the IRC calculation, suggesting the products should change compared with the naked reaction and the reactions with one additional participating H_2_O. In fact, owing to the direct participation of (H_2_O)_2_, the broken H migrates along the (H_2_O)_2_ skeleton, resulting in the formation of NF_2_O + (H_2_O)_2_-HF, as exhibited in Figure 4, which is different from the S_N_2 mechanism of the NF_3_ + OH reaction with one additional H_2_O. It is interesting that the formation of NF_2_O + (H_2_O)_2_-HF is exothermic by 70.49 kcal/mol. In a word, when two additional H_2_O molecules take part in the reaction of NF_3_ + OH as catalyst, the formation of the products NF_2_O + (H_2_O)_2_-HF is favorable both thermodynamically and kinetically.

Considering the F abstraction mechanism, a pre-reactive complex, **W2-RC2,** was confirmed as well. Due to the hydrogen bond, **W2-RC2** is 17.70 kcal/mol lower than the reactants, which is of marginal difference with the naked reaction and the reaction with one participating H_2_O molecule. The F abstraction was accomplished through **W2-TS2**, which is similar to **W1-TS2** as well. However, the energy barrier of **W2-TS2** is almost the same as that of **TS2** and **W1-TS2**, inferring that (H_2_O)_2_ has marginal influence on the F abstraction mechanism. This result is not unexpected because the (H_2_O)_2_ plays the role of spectator, as can be seen from the structure of **W2-TS2**.

Apart from the S_N_2 mechanism and F abstraction mechanism, there is a new reaction process in the case of (H_2_O)_2_ participating in the NF_3_ + OH reaction, as depicted in Figure 4. This process initiates by the formation of **W2-RC3**, which is a pre-reactive complex formed by the contact between (H_2_O)_2_-OH and NF_3_. Starting from **W2-RC3**, the reaction proceeds via the transition state of **W2-TS3**, in which the change in O-N and F-N bonds is similar to that in **W2-TS1**. It is interesting that the IRC calculation of **W2-TS3** bears evidence of the interaction between the substituted F and (H_2_O)_2_, leading to the formation of complex NF_2_O-(H_2_O)_2_-HF (see **W2-PC3** in Figure 4). It is worth noting that **W2-TS3** lies 0.53 kcal/mol below the reactants, suggesting this process in kinetically favorable as well.

**Figure 5:**
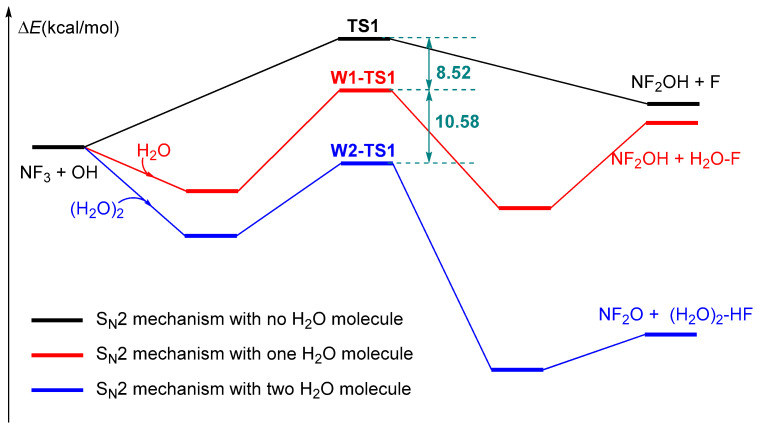
Comparison of naked NF_3_ + OH reaction and the H_2_O and (H_2_O)_2_ assisted reactions.

It is well-known that the concentrations of larger complexes involving more than two molecules are very low in the troposphere [37]; as a result, only an additional one or two H_2_O molecules were taken into account. In summary, it is obvious that the participation of additional H_2_O molecules influences the reaction of NF_3_ with OH dramatically. Taking the S_N_2 mechanism, for example (see Figure 5), without the assistance of additional H_2_O molecules, the reaction is difficult to accomplish, as the energy barrier is high, and the products are endothermic. Fortunately, the energy barrier of the S_N_2 mechanism decreases by 8.5 kcal/mol and 10.6 kcal/mol in the case of H_2_O and (H_2_O)_2_ catalyzed reactions. Especially, the thermodynamics of the reaction change as the products change from NF_2_OH + F to NF_2_O + HF with the formation of an O-H∙∙∙F hydrogen bond.

## 3. Materials and Methods

### Computational Methods

All reactants, products, pre-reactive complexes (RC, PC) and transition states (TS) were fully optimized using the density functional theory at the ωB97XD/aug-cc-pVTZ level of theory, as the long-range correction functional ωB97XD described the hydrogen bond well [51,52,53,54,55]. The harmonic vibrational frequencies of all optimized structures were calculated at the same level of theory to confirm the stationary point (intermediate or transition states) and for the zero-point energy (ZPE) corrections. The intrinsic reaction coordinate (IRC) [50] calculations were carried out to verify that the predicted transition states connect the designated reactants and products. In order to obtain more accurate thermodynamics data, the single-point energies of all species were calculated using the CBS-QB3 method [56,57]. The energy calculated at the CBS-QB3 level of theory was employed in the following discussion. All the DFT calculations were performed using the Gaussian 09 program [58]. The bond length comparison of selected species and all the optimized cartesian coordinates of species involved in the reactions are available in the Supporting Information (SI). The zero-point energy (ZPE) and relative energies are listed in Table 1. The energy profiles and corresponding structures for the reaction of NF_3_ with OH with the assistance of water molecules are illustrated in Figure 1, Figure 2, Figure 3, Figure 4 and Figure 5.

## 4. Conclusions

The possibility of removal of NF_3_ by the NF_3_ + OH reaction was studied at the CBS-QB3 level of theory. It was found that the NF_3_ + OH reaction in the absence of H_2_O molecules (naked reaction) is of no importance for the removal of NF_3_ in atmospheric conditions, as both the S_N_2 and F abstraction mechanisms must overcome a high energy barrier, while the products are endothermic. Although the participation of one H_2_O molecule has no influence on the NF_3_ + OH reaction, as the H_2_O acts as a spectator, it significantly changes when (H_2_O)_2_ takes part in the reaction as catalyst. The presence of (H_2_O)_2_ not only reduces the energy barrier of the S_N_2 mechanism, but also changes the products, i.e., with the formation of NF_2_O + (H_2_O)_2_-HF instead of NF_2_OH + (H_2_O)_2_-F. The reaction of NF_3_ + OH is favorable in the presence of (H_2_O)_2_, both kinetically and thermodynamically. The results indicate that it is possible to remove NF_3_ by reaction with OH radical in the presences of water molecules.

## Figures and Tables

**Table 1:** The ZPE and calculated relative energies (in kcal/mol) for all reactants, transition states and products.

Species	ZPE (ωB97XD/avtz)	Δ*E* + ZPE(ωB97XD/avtz)	Δ*E* (CBS-QB3)
NF_3_+ OH + *n*H_2_O	/	0	0
**TS1**	13.72	19.77	16.04
**TS2**	12.69	32.24	32.72
**W1-RC1**	28.24	−4.40	−7.12
**W1-RC2**	28.21	−4.32	−7.32
**W1-TS1**	29.58	13.87	7.52
**W1-TS2**	28.00	27.75	26.30
**W1-PC1**	31.23	−18.94	−18.84
**W1-PC2**	29.58	2.12	−0.53
**W2-RC1**	44.22	−9.60	−15.23
**W2-RC2**	44.88	−11.16	−17.70
**W2-RC3**	44.86	−11.28	−17.87
**W2-TS1**	45.73	5.99	−3.06
**W2-TS2**	44.55	20.69	15.78
**W2-TS3**	45.60	8.39	−0.53
**W2-PC1**	45.98	−66.14	−72.93
**W2-PC2**	45.56	−4.78	−10.50
**W2-PC3**	46.30	−66.01	−73.08
NF_2_OH + F	15.06	2.79	2.50
NF_2_ + HFO	12.84	8.95	10.02
NF_2_OH + H_2_O-F	28.39	5.95	1.90
NF_2_ + H_2_O-HFO	28.57	2.98	2.05
NF_2_O + (H_2_O)_2_-HF	43.85	−64.63	−70.49
NF_2_ + (H_2_O)_2_-HFO	44.99	−3.90	−7.92

## Data Availability

Data are contained within the article.

## Supplementary Materials

The following supporting information can be downloaded at: https://www.mdpi.com/article/10.3390/molecules29174033/s1, Figure S1: The optimized geometries of all species in the reaction of NF_3_ + OH with additional water molecule. Figure S2: IRC results for **W2**-**TS1**. Figure S3: IRC results for **W2**-**TS3**. Figure S4: Bond length comparison of selected species optimized at the level of ωB97XD/aug-cc-pVTZ (black) and MP2/aug-cc-pVTZ (red) methods. Optimized Cartesian coordinates of intermediates and transition states involved in Figure 1, Figure 2, Figure 3, Figure 4 and Figure 5.
