# Supplementary material for: Unveiling the Influence of Water Molecules for NF3 Removal by the Reaction of NF3 with OH: A DFT Study
Source: Molecules. 2024 Aug 26;29(17):4033. doi: 10.3390/molecules29174033 (PMC11396519; doi:10.3390/molecules29174033)
Supplement: Supplementary file 1 [file molecules-29-04033-s001.zip › molecules-3150398-supplementary.pdf]

Supporting Information

Unveiling the Influence of Water Molecules for NF_3 Removal by the Reaction of NF_3 with OH: A DFT Study

Jiaxin Liu [1,†], Yong Zhao [1,†], Xueqi Lian [2], Dongdong Li [1], Xueling Zhang [2], Jun Chen [1], Bin Deng [1], Xiaobing Lan [1,*] and Youxiang Shao [2,*]

[1] Hunan Provincial Key Laboratory of Xiangnan Rare-Precious Metals Compounds Research and Application, School of Chemistry and Environmental Science, Xiangnan University, Chenzhou 423000, China

[2] Key Laboratory of Electronic Functional Materials and Devices of Guangdong Province, School of Chemistry and Materials Engineering, Huizhou University, Huizhou 516007, China

* Correspondence: xblan@xnu.edu.cn (X.L.); shaoyx@whu.edu.cn (Y.S.)

[†] These authors contributed equally to this work.

1. Figure S1. The optimized geometries of all species in the reaction of NF_3 + OH with additional water molecule……………………………………………………1

2. Figure S2. IRC results of **W2-TS1**……………………………………….…………1

3. Figure S3. IRC results of **W2-TS3**……………………….……………………………1

4. Figure S4. The bond length comparison of selected species optimized at the level of ωB97XD/aug-cc-pVTZ(black) and MP2/aug-cc-pVTZ(red) methods……………..2

5. Optimized Cartesian coordinates ………………………….……………………….2

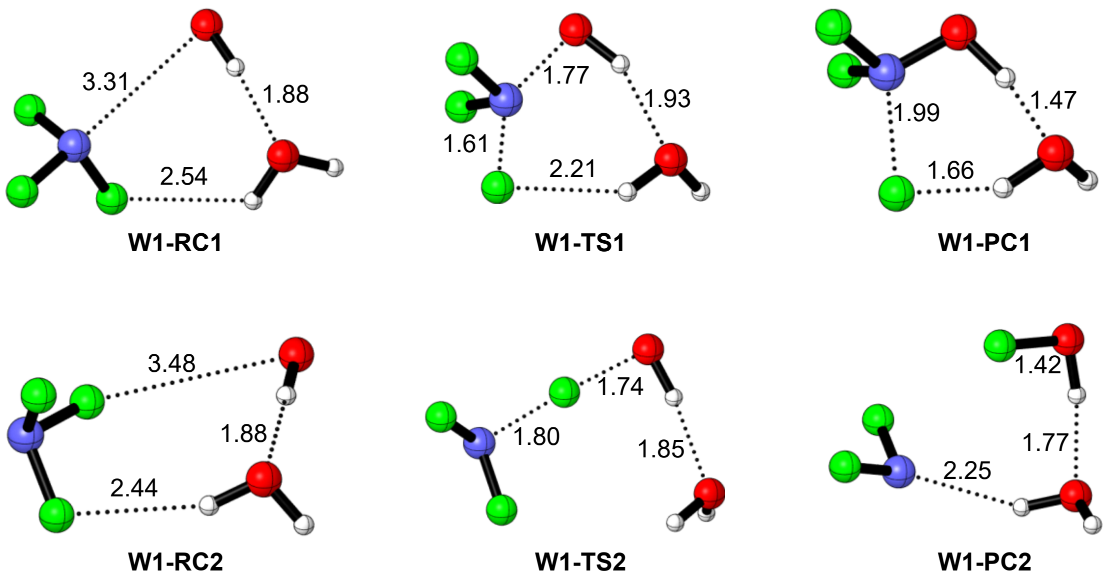

Figure S1. The optimized geometries of all species in the reaction of NF_3 + OH with additional water molecule. The distances are in Å.

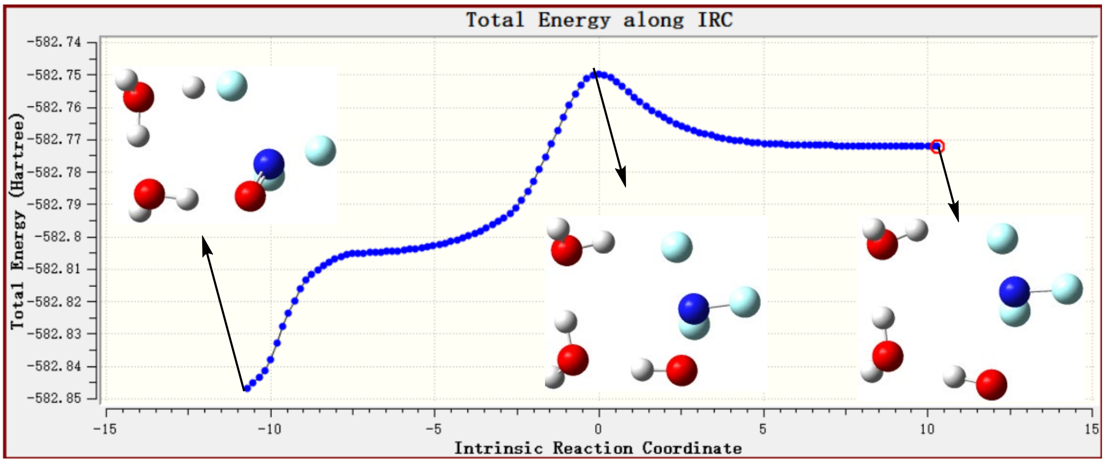

Figure S2. IRC results of **W2-TS1.**

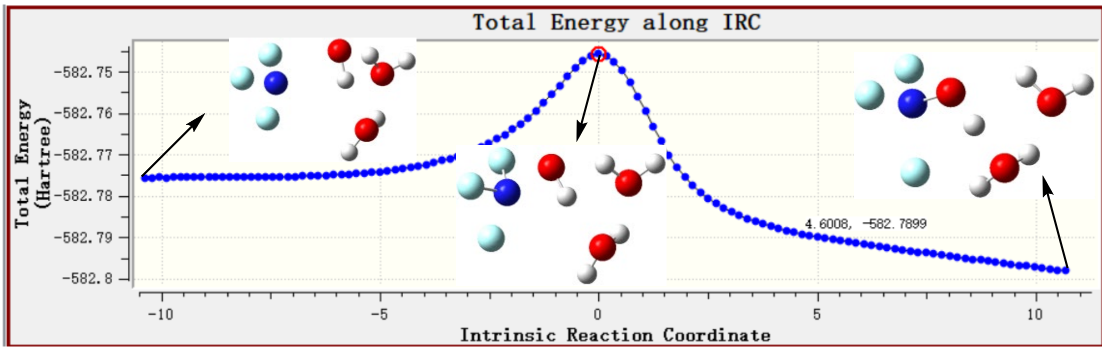

Figure S3. IRC results of **W2-TS3.**

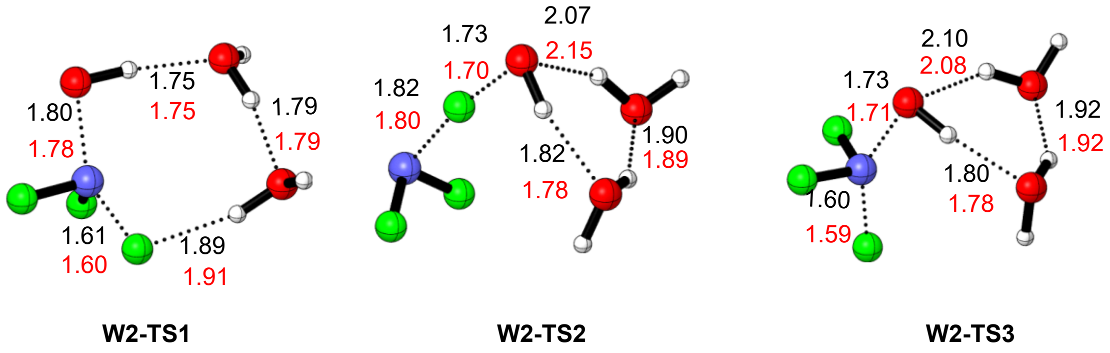

Figure S4. The bond length comparison of selected species optimized at the level of ωB97XD/aug-cc-pVTZ(black) and MP2/aug-cc-pVTZ(red) methods.

4. Optimized Cartesian coordinates.

TS1

N	0.0017234947	-0.0258964075	0.1004504904
F	1.473300303	-0.052469307	0.7392402646
F	0.0685350913	1.0934934959	-0.6045808896
F	0.1198796955	-0.9929593697	-0.8036928015
O	-1.6854678058	-0.0676447275	0.4430967968
H	-1.6365607786	-0.3636866842	1.3615061394

TS2

N	-0.956675487175	-0.000778147729	0.512434697801
F	-1.289708620661	1.056109033989	-0.218551388715
F	-1.342363848495	-1.030212915957	-0.229198727773
F	0.843616588134	-0.049561032853	0.204726679118
O	2.511674743963	-0.061119678328	-0.232351335351
H	2.779361624235	0.298682740858	0.624928074926

W1-RC1

N	0.9984867116	-0.0760419162	-0.2593003954
F	0.4366678687	-0.7003595319	0.8307915493
F	1.5898387763	1.0004852973	0.3065409186
F	2.0644939946	-0.8877958074	-0.5213298556
O	-2.5512841063	-0.9305971427	-0.2565157659
H	-1.9326070565	-1.4333981523	0.2752831415
H	-3.4243638605	-1.1743006051	0.0524602574
O	-1.7261969107	1.7984350581	-0.2415302373
H	-2.0572504173	0.8776728003	-0.2957976127

W1-TS1

N	0.5194785021	0.026607878	0.0308964139
F	1.1866747453	0.0515788439	1.1771530474
F	1.4610784195	0.1377127299	-0.893534575
F	0.1837978704	-1.5410128926	-0.074884189
O	-2.4743228391	-0.1243096537	-0.2413398226
H	-3.1646112346	-0.2665918503	0.4063673192
H	-1.936281095	-0.9217898023	-0.2318736622
O	-0.2456796243	1.626893532	-0.009987402
H	-1.1535987443	1.2720222152	-0.0883591296

W1-PC1

N	-0.60290200	-0.05765900	-0.00268700
F	-1.39348800	-0.03186900	1.10705300
F	-1.49298100	0.06830100	-1.01940600
F	0.62572100	1.51065900	0.00969900
O	2.34676200	-0.37074700	-0.09132800
H	2.87737800	-0.49514700	0.69666000
H	1.97186600	0.53891500	-0.05047600

O	-0.00848000	-1.20221600	-0.08722900
H	1.01155800	-0.98026300	-0.07502600

W1-RC2

N	-1.61772912	-0.09501693	-0.32152875
F	-1.02087621	-1.25665708	-0.76114198
F	-1.58080262	-0.25805676	1.02740501
F	-0.63694943	0.81745445	-0.53044185
O	2.10927527	-0.93862300	0.37323880
H	1.31078958	-1.25192404	-0.05442628
H	2.79450212	-1.56826810	0.14748922
O	2.65474766	1.83658308	-0.05207429
H	2.47564874	0.89048336	0.12832712

W1-TS2

N	-1.47805234	-0.25915927	-0.35386400
F	-1.06566180	-1.15597506	0.55214012
F	-2.07122308	0.67088932	0.38289106
F	0.08803484	0.56422679	-0.70491968
O	2.69000461	-0.92290135	0.51100092
H	2.08682949	-1.11743048	1.22995823
H	2.59314412	-1.64871408	-0.10734798
O	1.63194785	1.36672230	-0.76433467
H	2.07730932	0.61821583	-0.31528800

W1-PC2

N	-0.82905748	-1.00751805	-0.05738042
F	-1.58730914	-0.70108279	0.99532974
F	-1.41507591	-0.38651376	-1.07671173

F	0.96097269	1.57810892	0.59194456
O	2.29645442	-1.17384277	-0.09333884
H	2.73128337	-1.43174336	0.72054511
H	1.37734446	-1.43871994	0.00359545
O	1.98503973	1.53404258	-0.38387590
H	2.19691386	0.57590517	-0.32571998

W2-RC1

N	1.00613370	-0.42932277	-0.10995792
F	1.13185483	-0.06779253	1.18912815
F	2.27122415	-0.32397765	-0.57083722
F	0.85953885	-1.80861382	-0.00040783
O	-2.11549317	1.34784194	-0.00812432
H	-2.69521395	1.62734346	0.69946070
H	-2.16062313	0.37885848	-0.02997848
O	0.34827188	2.63891524	-0.30533513
H	-0.52821420	2.20133941	-0.21513110
O	-2.25091132	-1.46075038	-0.17516977
H	-2.63127268	-1.74265924	-1.00797213
H	-1.39922296	-1.89859615	-0.11767894

W2-TS1

N	0.95665436	-0.10357205	-0.03374155
F	1.22366728	0.03099755	1.26382007
F	2.13465272	-0.32493441	-0.58865881
F	0.39203829	-1.61019039	-0.02215951
O	-1.96935572	1.54025904	0.03762724
H	-2.28554471	1.87236763	0.87721855
H	-2.24375728	0.60548818	-0.00938296

O	0.69816422	1.59086993	-0.57656992
H	-0.26472544	1.64898493	-0.36093703
O	-2.40022794	-1.17115364	-0.12337776
H	-2.79652386	-1.52790119	-0.91832955
H	-1.49051191	-1.49560859	-0.11147976

W2-PC1

N	-1.56818026	-0.17636060	-0.07063826
F	-1.22622093	-0.32514192	1.28827925
F	-2.96908643	-0.09624482	-0.03240654
F	0.30951629	1.90966678	-0.43465609
O	1.86228385	-1.56459673	-0.07257052
H	1.77981484	-2.03730146	0.75649137
H	2.51989220	0.10138710	0.04614384
O	-1.10335689	-0.95412208	-0.81829763
H	0.96569493	-1.47851783	-0.40961861
O	2.66241461	1.06520842	0.08632756
H	3.32993989	1.27072745	-0.56819716
H	1.20826210	1.66890080	-0.23063821

W2-RC2

N	1.73360643	0.12727669	0.40573511
F	1.43582250	-1.09754459	-0.12453011
F	2.95893934	0.37681222	-0.14005727
F	0.92810405	0.95287912	-0.31890928
O	-1.72386100	-1.06738329	-1.13905713
H	-0.95139447	-1.15479169	-1.69721577
H	-1.40905188	-1.16407821	-0.22634668
O	-2.73500135	1.33595548	-0.21929190

H	-2.45926961	0.56812766	-0.77588916
O	-1.28916947	-0.43664070	1.54710756
H	-1.83217026	0.32290291	1.29081493
H	-1.68800229	-0.79913361	2.33760070

W2-TS2

N	-2.02556663	0.13443980	-0.46458112
F	-1.62250355	-1.10274532	-0.72871509
F	-2.28427471	0.10606009	0.84120743
F	-0.42346937	0.99576659	-0.44618399
O	1.76865617	-0.33979968	1.61698745
H	1.08000147	-0.77630633	2.11781501
H	1.91537184	-0.88220657	0.82430592
O	1.17854331	1.60900271	-0.23955872
H	1.32049467	1.09219041	0.58809533
O	1.99458675	-1.07160759	-1.06200330
H	1.73788947	-0.14660686	-1.18207067
H	2.82692459	-1.17422325	-1.52316525

W2-PC2

N	-2.16516308	-0.31564726	-0.18068230
F	-1.31855179	-1.35531217	-0.25565279
F	-1.92012473	0.19481606	1.03371430
F	0.52383804	1.29446222	-0.98312200
O	1.40122338	-0.47611171	1.42433695
H	0.60441726	-0.92645206	1.70595045
H	1.70499085	-0.93840909	0.62273248
O	1.08162914	1.87187050	0.18914124
H	1.15680667	1.04804494	0.74046506

O	2.08733777	-1.14747832	-1.15862040
H	1.66448970	-0.33329657	-1.45279896
H	2.99650477	-1.09072654	-1.45348600

W2-RC3

N	-1.07348242	-0.04080878	0.07298025
F	-1.70971417	1.15668826	0.13414594
F	-2.01213720	-0.89442823	0.57105116
F	-1.11508182	-0.32232271	-1.26454494
O	2.02802890	-1.26966309	-0.68489772
H	1.99613087	-0.33519662	-0.94581671
H	1.42530594	-1.73174769	-1.26692905
O	1.44425354	-0.24347295	1.82471823
H	1.61982204	-0.84102484	1.05936117
O	1.74342555	1.48920795	-0.45537131
H	2.38581787	2.19622140	-0.50810981
H	1.67670710	1.24943425	0.48048363

W2-TS3

N	1.01902534	0.18334361	0.01680402
F	2.30849958	0.15065783	0.33389896
F	0.85929611	-0.84556268	-0.80940245
F	0.38042309	-0.42419973	1.35197077
O	-1.15719010	2.72573301	0.14970405
H	-0.49035159	3.26993606	0.59805720
H	-1.56010673	2.18878148	0.83318516
O	0.99459642	1.53484480	-1.06968329
H	0.10531290	1.87346037	-0.80829609
O	1.35577837	3.72075553	0.84702346

H	1.78374931	4.55486564	0.65512440
H	1.65370743	3.10019592	0.16983001

W2-PC3

N	-1.47970391	-0.03193321	0.10578160
F	-0.75177918	-1.07096055	-0.52122919
F	-2.74181225	-0.63177982	0.32029790
F	1.14960182	0.43565997	1.57087288
O	1.85652028	1.36796074	-0.67916578
H	2.24561799	0.49964376	-0.88414730
H	1.36358605	0.93288973	0.78141079
O	-1.47176819	1.01130863	-0.42420587
H	1.13939947	1.50022068	-1.30067482
O	2.61650924	-1.24872660	-0.29609961
H	3.50217952	-1.53122213	-0.06764833
H	2.15663158	-1.12317121	0.54244271

NF_3

N	0.16901388	-0.00197817	-0.06235200
F	0.76089384	1.22344623	0.01892192
F	0.97145812	-0.64818595	-0.95513403
F	0.53550716	-0.57504311	1.11904311

W2-HF

F	1.10296900	0.38711500	1.57891200
O	1.86924100	1.35208500	-0.63767400
H	2.22704000	0.47529500	-0.86263700
H	1.34431000	0.89693000	0.80553000
H	1.16444600	1.52865100	-1.26226700

O	2.52444300	-1.30134700	-0.31926400
H	3.39614900	-1.62383000	-0.09004800
H	2.06068700	-1.18092600	0.51789500

W2-OH

O	-2.06623827	1.69857091	0.18001216
H	-2.25105873	2.04068658	1.05410199
H	-2.36885804	0.77593038	0.17375725
O	0.30017930	0.61006873	-0.75613029
H	-0.39662709	1.21970718	-0.41439282
O	-2.00880512	-1.05630570	-0.17634418
H	-2.42320555	-1.58788641	-0.85549975
H	-1.12142651	-0.84712166	-0.50102436

NF_3OH

N	-0.06385637	0.00001102	0.00860843
F	-0.35506417	1.06806153	-0.80866258
F	1.81185857	-0.00160041	0.74082631
O	-0.94350995	-0.00120375	0.99581760
F	-0.35565739	-1.06548788	-0.81176958
H	-1.83333357	-0.00040454	0.60283718

NF_2OH

N	-0.55088163	0.00195560	-0.30631562
F	-1.33596694	1.12383976	-0.11187765
F	-1.50013102	-0.99661895	-0.18507331
O	0.16185107	-0.09302019	0.84525775
H	1.07143952	-0.15278922	0.53542583

H_2O-F

F	0.04323900	1.64918000	0.00000000
O	0.04323900	-1.56284000	0.00000000
H	-0.89964100	-1.72626000	0.00000000
H	0.16456900	-0.61364000	0.00000000

H_2O-HFO

F	-1.31415100	-0.59733800	0.00809800
O	1.81137500	0.00191900	-0.01176000
H	2.03510700	-0.45861800	0.79847400
H	1.95415200	-0.63079900	-0.71747700
O	-0.84626200	0.73727300	-0.00652300
H	0.11719900	0.55192100	-0.00761000

HFO

F	-1.3139427719	-0.5942706691	0.0080670252
O	-0.8394412107	0.7316560585	-0.0064881106
H	0.1101699826	0.5544706105	-0.0076139146
